# The impact of shift-work light conditions on tissue-specific circadian rhythms of canonical clock genes: insights from a mouse model study

**DOI:** 10.12688/f1000research.136998.2

**Published:** 2023-08-02

**Authors:** Bala S. C. Koritala, Panshak P. Dakup, Kenneth I. Porter, Shobhan Gaddameedhi

**Affiliations:** 1 Department of Otolaryngology-Head and Neck Surgery, University of Cincinnati, Cincinnati, Ohio, USA; 2 Division of Pediatric Otolaryngology-Head and Neck Surgery, Cincinnati Children’s Hospital Medical Center, Cincinnati, Ohio, USA; 3 Department of Pharmaceutical Sciences, Washington State University, Spokane, Washington, USA; 4 Department of Biological Sciences, North Carolina State University, Raleigh, North Carolina, USA; 5 Center for Human Health and the Environment, North Carolina State University, Raleigh, North Carolina, USA

**Keywords:** Canonical clock genes, mouse, rotating shift, jet-lag, liver, skin.

## Abstract

**Background:** The natural day-night cycle synchronizes our circadian rhythms, but modern work practices like night shifts disrupt this pattern, leading to increased exposure to nighttime light. This exposure is linked to various health issues. While some studies have explored the effects of night shifts on human circadian rhythms, there is limited research on the consequences of long-term exposure to shift-work light conditions. Rodents can provide valuable insights into these effects. This study aimed to examine how short- or long-term exposure to rotating shifts and chronic jetlag affects the core circadian oscillators in the liver and skin of mammals.

**Methods:** C57BL/6J male mice were subjected to simulated shift-work light conditions, including short-term or long-term rotating shifts and chronic jet-lag conditions. Liver and skin samples were collected every four hours over a 24-hour period on the second day of constant darkness. RNA was extracted and qRT-PCR analysis was conducted to measure the circadian gene expression in liver and skin tissues. Circadian rhythm analysis using CircaCompare compared the control group to mice exposed to shift-work light conditions.

**Results: **The liver's circadian clock is significantly altered in mice under long-term rotating shift conditions, with a lesser but still noticeable impact in mice experiencing chronic jetlag. However, short-term rotating shift conditions do not significantly affect the liver's circadian clock. Conversely, all three simulated shift conditions affect the skin's circadian clock, indicating that the skin clock is more sensitive to shift-work light conditions than the liver clock. Compared to the liver, the skin's circadian clock is greatly affected by long-term rotating shift conditions.

**Conclusions: **The study findings indicate more pronounced disturbances in the canonical clock genes of the skin compared to the liver under simulated shift-work light conditions. These results suggest that the skin clock is more vulnerable to the effects of shift-work.

## Introduction

The natural day-night cycle is the primary synchronizer of circadian rhythms in humans. ^1^ The advent of electricity has led to 24/7 work practices including night shift and rotating shift schedules, increasing nighttime light exposure in modern society. Unfortunately, these modern work habits have been associated with an elevated risk of various health conditions, including cancer, cardiovascular diseases, depression, diabetes, gastrointestinal problems, and metabolic syndrome. ^2^^–^^5^ The disruption of circadian rhythms, both at the physiological and behavioral level, contributes to these modern work habit-associated health conditions. The few studies investigating the impact of night shift on the molecular circadian rhythms in humans have revealed significant changes in the circadian transcriptome and metabolome within a one-week study duration. ^6^^–^^8^ However, in real-world scenarios, many shift workers experience prolonged exposure to rotating shifts that extend beyond a single week. Unfortunately, there is a lack of systematic research investigating the consequences of long-term exposure to shift-work light conditions on molecular circadian rhythms in humans. This absence may be attributed to logistical, financial, and facility constraints in setting up human sleep laboratories. Nonetheless, rodents can serve as valuable preliminary models for understanding the effects of different shift schedules or exposure to shift-work light conditions on molecular circadian rhythms, providing insights into the underlying molecular mechanisms associated with shift-work related disorders. ^9^

In laboratory settings, researchers can replicate shift-work-like light environments using rotating shift and jet-lag protocols in rodents. Several studies ^10^^–^^14^ have employed these protocols in rodents to investigate the mechanisms underlying tumor development, metabolic disorders, and other health outcomes associated with shift work. These studies have indicated that exposure to shift-work or jet-lag light environments can promote tumor progression, ^10^^,^^12^^,^^15^^–^^18^ glucose intolerance, ^19^^,^^20^ obesity, ^19^^,^^21^ genotoxic stress mediated cardiovascular toxicity, ^22^ and radiodermatitis. ^23^ Although some research has shown abnormal activity/sleep rhythms in response to shift conditions, ^11^ our understanding of how short- or long-term exposure to shift-work and chronic jet-lag conditions affects the mammalian core circadian oscillator, the canonical clock genes, remains limited. Rodents serve as a convenient model for studying tissue-specific circadian gene expression of these clock genes. Therefore, in this study, we utilized a well characterized circadian mouse model, C57BL/6J, ^24^ to investigate the impact of short- or long-term exposure to rotating shifts and jet-lag conditions on the mammalian core oscillators in various tissues.

The primary focus of our study was to examine the effects of shift-work-like light conditions on the liver and skin. We specifically chose the liver because it is widely recognized as the primary organ model for studying the circadian clock in peripheral tissues. ^25^ The liver plays a crucial role in carrying out various metabolic functions regulated by the circadian clock through transcriptional and posttranslational mechanisms. ^26^^–^^28^ On the other hand, although the skin is not as metabolically active as the liver, it serves as accessible window for understanding the body’s internal clock in humans. ^29^^,^^30^ Moreover, a study has shown that restricted feeding altered the phase and amplitude of clock genes in mouse skin, subsequently affecting the skin’s response to ultraviolet radiation exposure. ^31^ Given these factors, the liver and skin represent distinct metabolic tissue models with robust circadian clocks, offering valuable insights into circadian rhythm disturbances caused by shift work.

**Figure 1.**
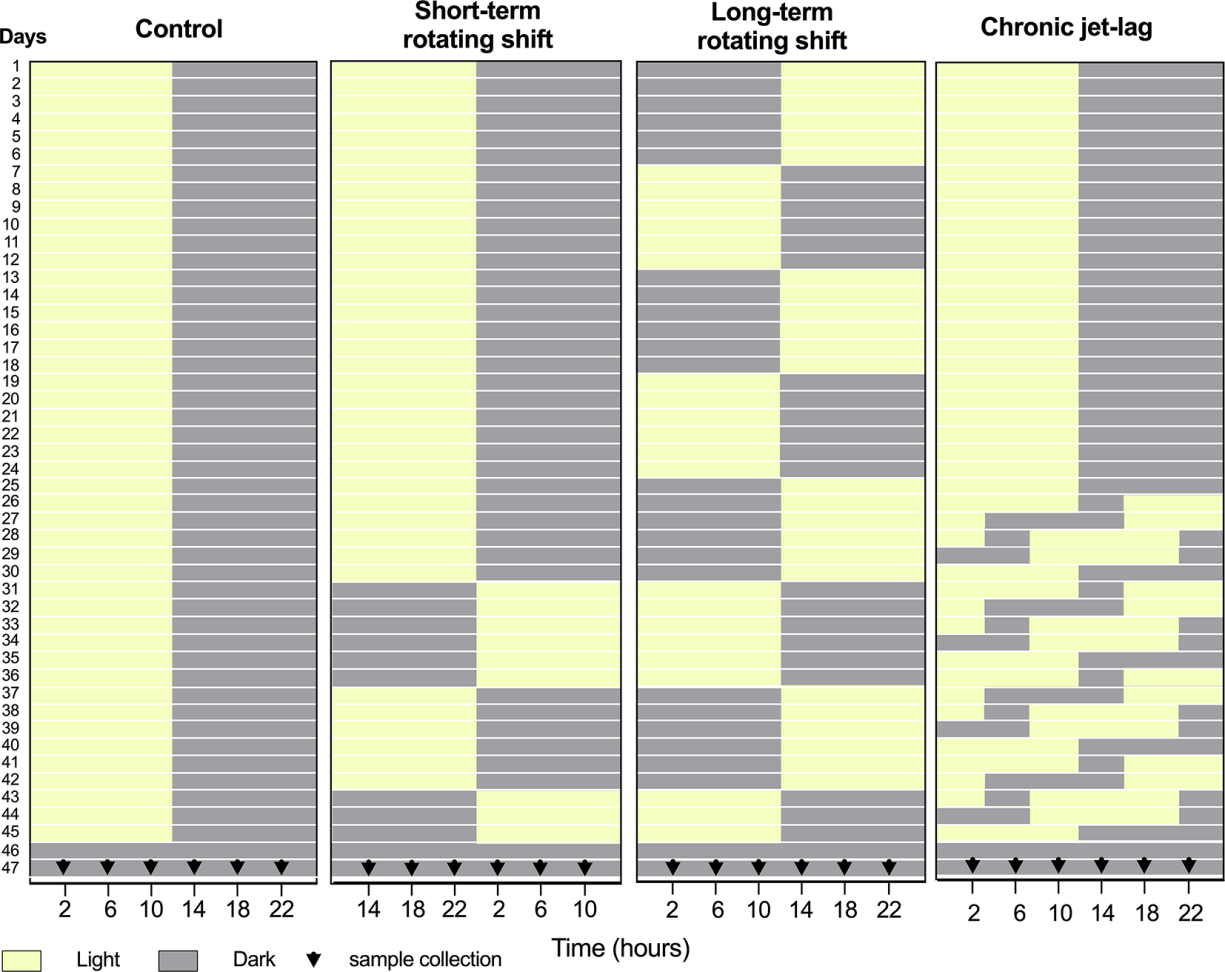
Design of simulated shift-work light conditions for mice. Panels from left to right represent simulated light conditions of control (left), short- and long-term rotating shifts (middle), and chronic jet-lag (right). After exposure to simulated shift conditions of light (yellow) and dark (Gray), liver and skin samples were collected for 24 hours with four-hour intervals on the second day of constant dark condition. 0 ^th^ time was defined as the onset of the light phase before the animals entered constant darkness for tissue collection.

Our study employed a comprehensive design that enabled us to investigate the tissue-specific disturbances in circadian rhythms resulting from simulated short- or long-term exposure to rotating shifts and chronic jet-lag conditions. After subjecting C57BL/6J mice to simulated shift-work light conditions, we measured the circadian gene expression of canonical clock genes ( *Arntl, Npas2, Per1, Per2, Per3, Cry1, Cry2,* and *Dbp*) in liver and skin samples. The measurements were taken over a 24-hour period on the second day of constant darkness ( Figure 1). Our findings indicate more pronounced disturbances in the canonical clock genes of the skin compared to the liver under simulated shift-work light conditions. Notably, long-term rotating shift work significantly impacted the expression of these canonical clock genes in both tissues. Based on our observations, it is evident that the skin clock is more vulnerable to the effects of shift-work light conditions when compared to the liver clock.

## Methods

### Ethics statement

The mice utilized in this study were procured from The Jackson Laboratory and housed in the Program of Laboratory Animal Resources (PLAR) of Washington State University - Spokane. To guarantee proper animal welfare, the guidelines set by the Institutional Animal Care and Use Committee (IACUC) were followed. Our program compiles with all regulations outlined in the NIH guide for the Care and Use of Laboratory Animals, encompassing procurement, conditioning and quarantine, housing, management, veterinary care, and carcass disposal. All efforts were made to ameliorate the suffering of the mice involved. There were no adverse events during the study and no humane endpoints were established. The animal experiments conducted in this study were approved by the IACUC under the protocol number: 6202 on March 27 ^th^, 2019.

### Study design and sample collection

This investigation took place from May through September 2019. Healthy immunocompetent wild-type C57BL/6J male mice at 3 weeks of age were purchased from the Jackson Laboratory (strain #: 000664). Mice were purchased to limit variability and maintain consistency in the genetic quality of the animals. Upon arrival, these mice were acclimatized to the controlled environment of the PLAR facility at Washington State University – Spokane for at least one week. The only inclusion criterion was for animals to be at least 4 weeks of age by day 1. To investigate the impact of shift-work light conditions on circadian rhythms of canonical clock genes, a group of C57BL/6J male mice were randomly assigned and housed (3 mice per cage) in a controlled environment that simulated shift-work light conditions. The mice were subjected to different simulated conditions: either short-term (15 days) or long-term (45 days) rotating shifts, where the light conditions changed every seventh day, or a jet-lag condition lasting 21 days with an 8-hour phase advance every two days. As a comparison, a control group of mice followed the standard light-dark cycle of the facility ( Figure 1). A total of 72 mice were included in this study, to cover 24 timepoints across 4 light conditions. An n = 3 was chosen per time point as the minimum number required to identify statistical differences per time-point, which translates to n = 18 per experimental condition for circadian analyses. Following exposure to simulated light conditions with an intensity of ~590 lux, the mice were housed in constant dark for up to 48 hours. On the second day, mice were sacrificed at the age of 11 to 12 weeks old, and samples of liver and skin tissues were collected from three mice at each time point over a 24-hour period, with four-hour intervals ( Figure 1). Specifically, whole skin tissue was collected from dorsal lateral side of the mouse following post-cervical dislocation, placed on fixed card stock, covered with foil, snap-frozen, and stored at -80 °C for RNA extraction. To manipulate the light conditions, the mice were housed in light-tight circadian chambers provided by Phenome Technologies, Inc. The light schedules were regulated by an external timer (XT-4, Chrontrol Corporation), and the light status and intensity were monitored using HOBO data loggers (UA-002-08, Onset Computer Corporation). All mice, regardless of the light conditions or constant darkness, were given *ad libitum* feeding. Throughout the time-course sample collection, red light was utilized, and animals were killed per housed group, where one cage of 3 mice represented one time-point. This was done to limit variability in any time-point. Mice were anesthetized with 3-4% isoflurane and euthanized by cardiac puncture blood collection followed by cervical dislocation as a secondary method of euthanasia. The collected liver and skin tissues were snap-frozen and stored at -80 °C for subsequent RNA extraction.

### Gene expression analysis

Frozen liver and skin tissues were homogenized in liquid nitrogen using mortar and pestle. Next, we purified the total RNA from the homogenized tissues using a Trizol-based RNA extraction protocol. Approximately 100 mg of the ground tissue was combined with 1 ml of ice-cold TRIzol reagent (Thermo Fisher Scientific, #15596018). After thorough mixing, 0.2 ml of chloroform was added to the TRIzol tissue cocktail, mixed well, and incubated at room temperature for 3 mins. We separated the different phases of the mixture through centrifugation at 12,000 g for 15 mins. The aqueous phase (top layer) was carefully collected and transferred to a new tube. To precipitate the RNA, an equal volume of isopropanol was added to the collected aqueous phase and mixed by inverting. After a 10-minute incubation at room temperature, the samples were subjected to another round of centrifugation at 12,000 g for 10 minutes. The supernatant was discarded, and the RNA pellet was washed three times with 70% ice-cold ethanol. Subsequently, the RNA pellet was air dried for 5-10 mins and dissolved in 20 μl of ddH _2_O. To assess the quantity and quality of the extracted RNA, we utilized NanoDrop ^®^ 2000 Spectrophotometer, measuring the light absorbance at 260, 280 and 230 nm. Following the RNA purification step, we treated the RNA samples with DNase-I (New England Biolabs, #M0303S), and synthesized complementary DNA (cDNA) using TaqMan™ Reverse Transcription Reagents (Thermo Fisher Scientific, #N8080234). Real-time polymerase chain reaction (RT-PCR) was then performed using PerfeCTa ^®^ SYBR ^®^ Green Supermix with ROX™ (Quantabio, #95055-500) in a StepOnePlus™ Real-Time PCR System (Applied Biosystems™). The RT-PCR reaction included denaturation (3 minutes at 95 °C) and 40 PCR cycles (15 sec at 95 °C and 1 minute at 60 °C). The expression level of each clock gene was normalized to the geometric mean expression of two housekeeping genes, ^32^ *Actb* and *Ppib* using the ΔCt method. Subsequently, the data were scaled to the sample with the highest expression level (ΔΔCt) for the time course analysis. Missing values in the raw data files resulting from technical issues with the qPCR plate were excluded from analysis. We specifically measured the expression of canonical clock genes ( *Arntl, Npas2, Per1, Per2, Per3, Cry1, Cry2,* and *Dbp*) in both liver and skin tissues using validated primers. To ensure the accuracy of the PCR amplification, the primers were validated using cDNA template serial dilution, and their efficiency was calculated based on the slope value ^33^ ( Table 1). The primer design for this study was performed using Primer3 (v.0.4.0), a widely used primer design tool.

**Table 1.** Primers used for measuring mRNA expression of canonical clock genes.

Gene	Forward Primer (5′ to 3′)	Reverse Primer (3′ to 5′)
*Arntl*	CTCAACCATCAGCGACTTCA	CCTTCCTTGGTGTTCTGCAT
*Npas2*	CCTAGCCCCTCCTGTAATGG	AGGTTCGTCAGCTACACACA
*Per1*	CCAGGATGTGGGTGTCTTCT	GACCCGAATCTTGGTCACAT
*Per2*	TGGTTTCTGGGAAGATCCTG	AGCTGTGGAACACACTGACG
*Per3*	CACCCTCCGTTTGAACACTC	GGCTCCAGGGGTTGACAAA
*Cry1*	CAGATCCCTTGGGACAAGAA	TGAGTCATGATGGCGTCAAT
*Cry2*	GGTGACCCGTTTGACCTTTG	TTCTCAGTCACCACCTCCAC
*Dbp*	GCAGGCTTGACATCTAGGGA	CTCATGGCCTGGAATGCTTG
*Actb*	CATTGCTGACAGGATGCAGAAGG	TGCTGGAAGGTGGACAGTGAGG
*Ppib* ^52^	GGAGATGGCACAGGAGGAAA	CCGTAGTGCTTCAGTTTGAAGTTCT

### Circadian rhythm analysis

To analyze the differences in circadian rhythms of canonical clock genes in animals exposed to both control and simulated shift-work light conditions, we utilized the standard parameters of the CircaCompare R package with a 24 hour fixed period. ^34^ Circadian time, CT0 was defined as the onset of the light phase before the animals entered constant darkness for tissue collection. This alignment allowed us to effectively compare and assess the variations in circadian rhythms between control and simulated shift-work light conditions. We applied a p-value cut-off of *<* *0.05* to determine the presence or absence of rhythms and to evaluate significant differences in phase, amplitude, and the Midline Estimating Statistic of Rhythm (MESOR). Sample resolution is a critical factor in circadian analysis as it directly affects the precision of phase estimates. ^35^ In our study, we used four-hour sample resolution, therein phase differences were only considered if they were at least four hours apart. This approach was implemented to ensure the accuracy of our results.

## Results

### Circadian analysis of canonical clock genes in the liver after exposure to simulated shift- work light conditions

**Figure 2.**
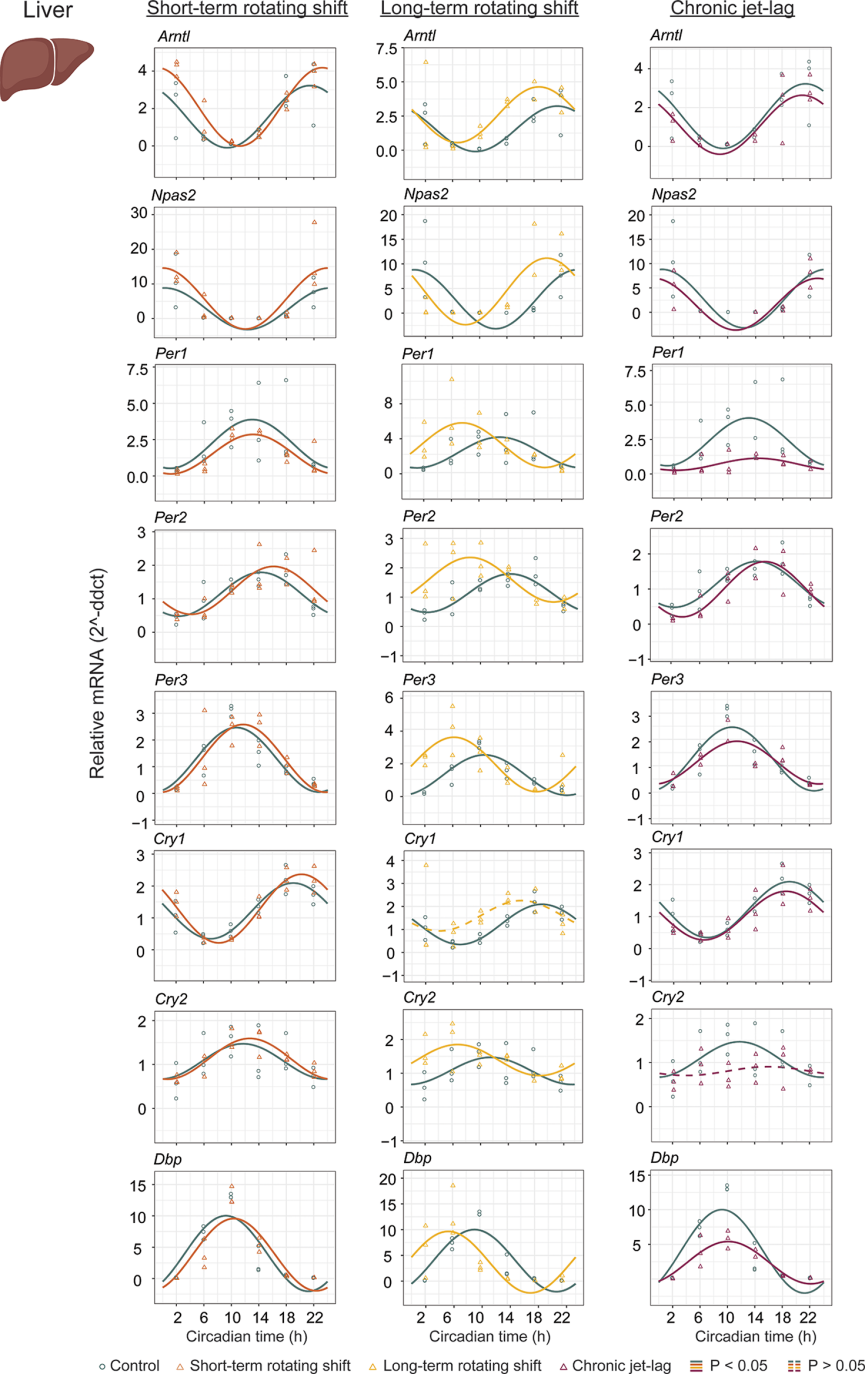
Circadian rhythms of canonical clock genes in the liver following exposure to simulated shift-work light conditions. The graphs display 24-hour expression (Mean ± SE) of *Arntl, Npas2*, *Per1, Per2, Per3*, *Cry1, Cry2,* and *Dbp* in the liver under constant dark after exposure to simulated shift-work light conditions. The figure allows for a comparison between the control group and three experimental conditions: short-term rotating shift (left), long-term rotating shift (middle), and control vs chronic jet-lag (right) were represented in the figure. To ensure accurate analysis, the mRNA expression throughout the time course was normalized against the geometric mean expression of housekeeping genes *Actb* and *Ppib.* The statistical significance of the circadian rhythms and the differences in circadian characteristics between the control group and the simulated shift-work conditions were assessed using CircaCompare, with a significance level set at *p < 0.05.* Genes that exhibit a significant rhythmic pattern are represented by continuous lines, while arrhythmic genes are represented by dotted lines.

To investigate the impact of shift-work light conditions on the liver clock, we examined the circadian rhythms of canonical clock genes ( *Arntl, Npas2, Per1, Per2, Per3, Cry1, Cry2,* and *Dbp*) in the liver samples of mice exposed to simulated shift-work light conditions. Rhythmic gene expression and its significance was determined at *p < 0.05* of CircaCompare. Our findings revealed significant rhythmic patterns in all analyzed clock genes, both in the control group and in the mice exposed to the simulated shift-work light conditions. However, *Cry1* did not show significant rhythmicity in the long-term rotating shift scenario and *Cry2* did not exhibit significant rhythmicity in the chronic jet-lag scenario ( Figure 2, Extended Data Table 1 ^54^). This suggests that these genes may be more sensitive to prolonged disruption of light-dark cycles. Furthermore, we examined various circadian properties such as phase, amplitude, and MESOR and compared differences between conditions ( Figures 2 and 4A, Extended Data Table 1 ^54^). In the short-term rotating shift and chronic jet-lag scenarios, we did not observe any discernible phase differences compared to the control condition. However, in the long-term rotating shift scenario, significant phase advance of at least four hours were detected in five out of eight clock genes: *Npas2*, *Per1, Per2, Per3,* and *Cry2.* Regarding amplitude, we did not find significant differences in the rotating shift conditions. However, we did observe a significant decrease in amplitude of *Per1* and *Dbp* among mice exposed to chronic jet-lag compared to the control group. This indicates that chronic disruption of light-dark cycles may affect the robustness of circadian rhythms in these genes. We did not find any significant changes in MESOR with short-term rotating shift. However, we observed a significant increase in MESOR with the genes *Arntl, Per2, Per3,* and *Cry2* in the long-term rotating shift condition. Conversely, we found a significant decrease in MESOR with the genes *Per1* and *Dbp* in the chronic jet-lag scenario. Overall, these results suggest that the circadian clock in the liver is highly impacted in mice exposed to the long-term rotating shift condition with a clear but less dramatic impact in mice exposed to chronic jet-lag. In contrast, we found no significant impact on the liver’s circadian clock in mice exposed to a short-term rotating shift condition.

### Circadian analysis of canonical clock gene expression in the skin after exposure to simulated shift-work light conditions

**Figure 3.**
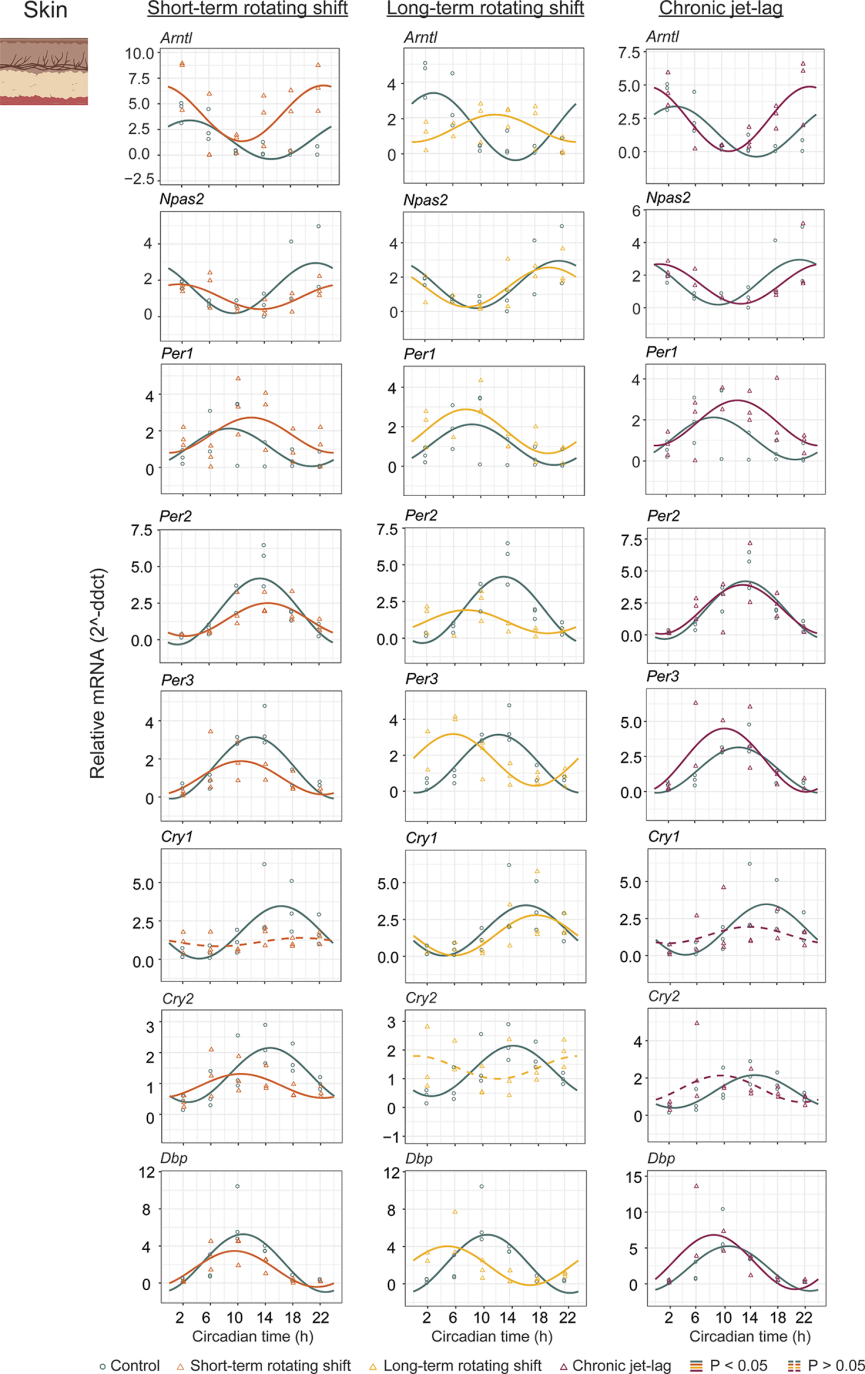
Circadian rhythms of canonical clock genes in the skin following exposure to simulated shift-work light conditions. The graphs in the figure illustrate the 24-hour expression (Mean ± SE) of *Arntl, Npas2*, *Per1, Per2, Per3*, *Cry1, Cry2,* and *Dbp* in the skin under constant darkness following exposure to simulated shift-work light conditions. The figure enables a comparison between the control group and three experimental conditions: short-term rotating shift (left), long-term rotating shift (middle), and control vs chronic jet-lag (right) were represented in the figure. To ensure precise analysis, the mRNA expression throughout the entire time course was normalized against the geometric mean expression of housekeeping genes *Actb* and *Ppib.* The statistical significance of the circadian rhythms and the variations in circadian characteristics between the control group and the simulated shift-work conditions were evaluated using CircaCompare, with a significance level set at *p < 0.05.* In the figure, genes that exhibit a significant rhythmic pattern are depicted by continuous lines, while genes lack a rhythmic pattern are represented by dotted lines.

**Figure 4.**
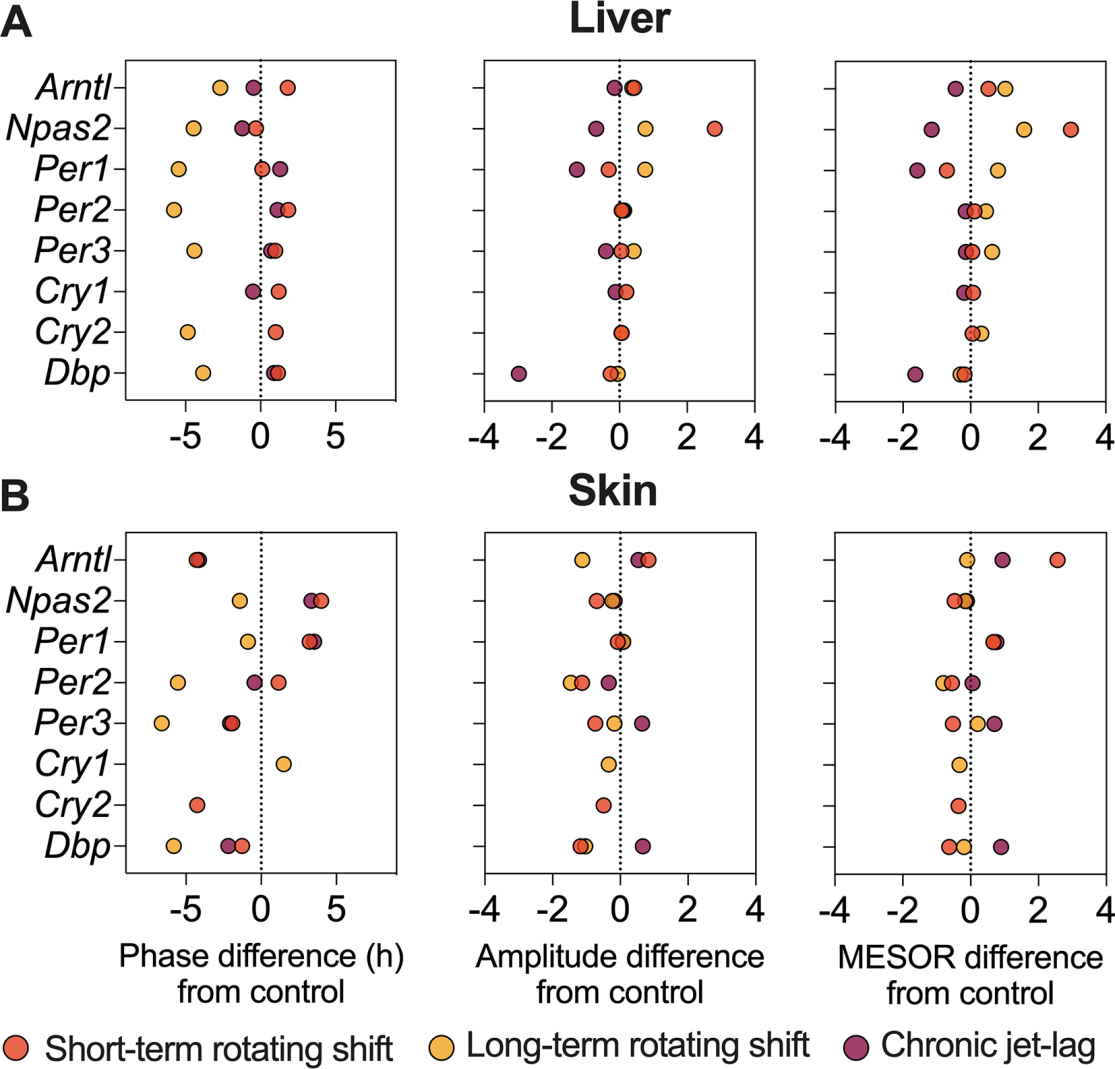
Differential circadian properties of canonical clock genes in the liver and skin following exposure to simulated shift-work light conditions. The figure represents differences in phase (left), amplitude (middle), and MESOR (right) of canonical clock genes in the liver (top) and skin (bottom) of mice subjected to short-term, long-term rotating shift, and chronic jet-lag conditions, in comparison to a control condition. Significance testing was performed using CircaCompare with a threshold of *p < 0.05*, and the corresponding p-values can be found in Extended Data Table 1. ^54^

In addition to studying the liver, we also examined the expression of canonical clock genes in the dorsal skin of mice ( Figure 3, Extended Data Table 1 ^54^) collected under constant darkness after being exposed to simulated shift-work light conditions. We analyzed the circadian rhythms and determined their significance using CircaCompare with a *p < 0.05.* Our analysis revealed that majority of the analyzed clock genes in the skin exhibited rhythmic patterns. However, we observed a loss of rhythmicity in *Cry1* after exposure to short-term rotating shift and chronic jet-lag conditions. Additionally, *Cry2* became arrhythmic when subjected to long-term rotating shift and chronic jet-lag conditions. Furthermore, we compared the circadian rhythms between the control group and the simulated shift-work groups, focusing on differences in phase (at least four hours), amplitude, and MESOR ( Figure 4B, Extended Data Table 1 ^54^). Out of the eight genes analyzed in the skin, significant phase differences were observed in two genes ( *Arntl* and *Cry2*) following the short-term rotating shift condition. Four genes ( *Arntl, Per2, Per3,* and *Dbp*) exhibited significant phase differences following the long-term rotating shift condition, and one gene ( *Arntl*) showed a significant phase difference following chronic jet-lag. Notably, most of these genes displayed a phase advance following simulated shift-work conditions compared to the control group. *Arntl* consistently exhibited dysregulation across all simulated shift conditions, particularly showing a phase delay in the long-term rotating shift scenario. Moreover, we observed alterations in amplitude: *Cry2* and *Arntl* exhibited reduced amplitude following short-term and long-term rotating shift conditions, respectively. *Per2* displayed reduced amplitude in both rotating shift conditions, while no significant differences in amplitude were noted with chronic jet-lag. Regarding MESOR, two out of eight genes analyzed in each shift condition exhibited MESOR differences compared to the control group. *Arntl* displayed a significant increase in MESOR following exposure to short-term rotating shift and chronic jet-lag conditions, while *Per1* showed a significant increase following long-term rotating shift and chronic jet-lag. Additionally, *Cry2* and *Per2* exhibited a significant decrease in MESOR following exposure to the short-term and long-term rotating shift conditions, respectively. Overall, these results indicate that the circadian clock in the skin is impacted by all three simulated shift conditions and suggest that the skin clock is more sensitive to shift-work light conditions compared to the liver clock. Similar to our observations in the liver, the circadian clock in the skin is highly impacted in mice exposed to long-term rotating shift conditions.

## Discussion

Our study aimed to fill a significant knowledge gap regarding the effects of different durations and types of shift conditions on the circadian system. Shift-work has been hypothesized to disrupt the synchronization between the central and peripheral circadian machinery. ^36^ Previous studies in humans have only examined the impact of mistimed sleep on molecular rhythms for about a week, ^6^^–^^8^ limiting our understanding of the long-term consequences. We conducted a study using a well-established C57BL/6J mouse model to investigate the consequences of various durations and types of shift-work light conditions on the circadian system. By simulating shift-work light conditions, we were able to observe changes in the circadian expression of canonical clock genes in both the liver and the skin.

Light is widely recognized as the primary synchronizer for maintaining the mammalian circadian system. Two photoreceptors, melanopsin (OPN4) and neuropsin (OPN5), have been identified as mediators of light entrainment from the retinal ganglion cells to the suprachiasmatic nucleus (SCN), ^37^^,^^38^ which serves as the central pacemaker of the circadian system. Interestingly, recent studies have shown that OPN5 in murine melanocytes is involved in circadian photoentrainment and the expression of clock genes, a phenomenon not observed in the liver. ^39^ On the other hand, the liver, being a metabolically active organ, can directly entrain its clock through feeding-fasting cycles. ^40^^–^^42^ Prior research has demonstrated this ability, indicating that the liver is more influenced by metabolic cues compared to the skin. Therefore, in the current study, we examined the impact of simulated shift-work light conditions on the circadian gene expression of canonical clock genes in two different tissues: the liver and the skin.

Unlike the skin, which directly senses external light stimuli, ^39^ the entrainment of liver clocks by light serves as a secondary event. In addition to synchronization from the central clock, feeding has been well demonstrated as a mechanism to reset liver clocks. ^40^^–^^42^ In our study, we provided mice with unrestricted access to food, allowing us to primarily observe changes arising from shift-work light conditions. The data from our study indicated that short-term exposure to rotating shift-light conditions did not significantly affect the circadian rhythms of liver clock genes. However, long-term exposure to rotating shifts resulted in circadian dysregulation, as evidence by loss of rhythmicity or significant differences in phase, amplitude, or MESOR in 7 out of 8 tested clock genes, and the jet-lag condition affected 3 out of 8 of the genes tested. These results suggest that liver clocks exhibit adaptability under less aggressive shift-work light conditions, possibly due to input from multiple environmental cues. Moreover, recent research has demonstrated that restricted feeding not only entrains liver clocks but also influences skin clocks in a distinct manner, highlighting an interesting interplay wherein a single stimulus can impact multiple systems. ^31^

In our investigation, we observed significant disturbances in the circadian rhythm of canonical clock genes in the skin under various shift-work light conditions. Short-term exposure to rotating shift schedules and chronic jet-lag led to the disruption of circadian rhythmicity in 4 out of 8 tested canonical clock genes, while long-term exposure to rotating shifts affected 6 out of 8 of the genes, with most of these changes attributed to phase differences. Notably, unlike the liver, the circadian clock in the skin exhibited significant dysregulation across all shift-work light conditions examined in our study. This suggests that the skin’s circadian clock is more susceptible to the impact of shift-work light conditions compared to the clock in the liver. One potential explanation for this susceptibility is the involvement of opsins-mediated signaling. Opsins are a class of light sensing G protein-coupled receptors that trigger signaling cascades upon photosensation. Previous studies have identified the expression of various opsins in different models, including murine melanocytes, ^39^ human melanocytes, keratinocytes, and dermal fibroblasts, and hair follicle stem cells. ^43^ The presence of opsins within the skin cells indicates the possibility of light-sensing capabilities in the skin tissue and the potential influence of environmental light cues on core clock regulation. However, experimental verification of this hypothesis is still required.

At the level of independent clock genes, we found that the circadian rhythms of cryptochrome genes ( *Cry1* and/or *Cry2*), which are the primary repressors of the molecular clock and also involved in maintaining genome integrity of cells, ^44^^,^^45^ were significantly disrupted under simulated shift conditions in both tissues. However, in the case of short-term rotating shift specifically affecting the liver, the rhythmicity of these genes remained intact. Cryptochromes (CRYs), found across various species from plants to animals, conserved evolutionarily, function in photoreception and signal transduction. ^46^ Their precise roles in mammalian circadian rhythm and light perception are complex, yet evidence suggests that CRYs may act as circadian photopigments, a possibility supported by experiments involving vitamin A depletion and mutations. ^47^^,^^48^ Our findings align with this research, indicating that *Cry* genes could be more sensitive to altered light shifts than other clock genes. Consequently, their rhythms may be uniquely susceptible to disruption under simulated shift conditions.

Moreover, our findings are consistent with existing literature in humans, which has associated disrupted rhythms of *Cry1* and *Cry2* with circadian dysregulation of DNA repair and increased DNA damage following exposure to simulated night shift conditions. ^49^ Additionally, we observed consistent dysregulation of the circadian transcription factor *Arntl*, which is involved in protecting skin cells and regulating UVB associated DNA repair response and melanin pigmentation. ^23^^,^^50^^,^^51^ This dysregulation occurred following exposure to all different types of simulated shift-work light conditions utilized in this study.

Our study has few limitations that should be considered when interpreting the findings. First, the use of a mouse model may limit the generalizability of the results to humans. Second, the simulation of shift-work light conditions may not fully capture the complexity of real-world shift schedules. Third, the sample size was relatively small, potentially affecting the statistical power. Fourth, the duration of exposure in our study may not fully reflect the long-term consequences of shift-work in humans.

Despite these limitations, our study emphasizes the importance of recognizing the varying impacts of different shift conditions on the circadian system. We highlight the heightened susceptibility of the skin to circadian misalignment under shift-work light conditions, in comparison to the liver. Furthermore, we emphasize the significance of tissue-specific responses and the role of light regulating circadian rhythms, specifically focusing on canonical clock genes during shift-work. These findings offer valuable insights that can guide future research, potentially leading to the discovery of novel mechanisms and more effective treatment strategies for shift-work associated diseases.

## Author contributions

**Bala S. C. Koritala:** Conceptualization, Data curation, Formal analysis, Investigation, Methodology, Resources, Software, Validation, Visualization, Writing – original draft preparation, Writing – review & editing. **Panshak P. Dakup:** Conceptualization, Investigation, Methodology, Resources, Validation, Writing – original draft preparation, Writing – review & editing. **Kenneth I. Porter:** Investigation, Methodology, Resources, Validation, Writing – review & editing. **Shobhan Gaddameedhi:** Conceptualization, Data curation, Funding acquisition, Methodology, Project administration, Resources, Supervision, Validation, Writing – review & editing.

## Data Availability

figshare: Raw data files with replicates titled as “Cycle threshold values of qPCR data for genes in the liver and skin of animals that were exposed to both control and simulated shift conditions”. https://doi.org/10.6084/m9.figshare.23269757.v1. ^53^ figshare: Extended Data Table 1. CircaCompare analysis of canonical clock genes in the mouse liver and skin following exposure to simulated shift-work light conditions. https://doi.org/10.6084/m9.figshare.23512155.v1. ^54^ Data are available under the terms of the Creative Commons Attribution 4.0 International license (CC-BY 4.0). figshare: ARRIVE checklist for ‘The impact of shift-work light conditions on tissue-specific circadian rhythms of canonical clock genes: insights from a mouse model study’. https://doi.org/10.6084/m9.figshare.23519379.v1. ^55^
