# Supplementary material for: The impact of shift-work light conditions on tissue-specific circadian rhythms of canonical clock genes: insights from a mouse model study
Source: F1000Res. 2023 Aug 17;12:762. Originally published 2023 Jun 28. [Version 3] doi: 10.12688/f1000research.136998.3 (PMC10422053; doi:10.12688/f1000research.136998.3)
Supplement: Peer review history

# Peer review: Reviewer response for version 3

**DOI:** 10.5256/f1000research.154609.r197687

**Published:** 2023-08-22

**Authors:** Brian J. Altman

**Affiliations:** 1 Department of Biomedical Genetics, Wilmot Cancer Institute, University of Rochester Medical Center, Rochester, NY, USA

I approve of all changes made to the revised manuscript.

Is the work clearly and accurately presented and does it cite the current literature?

Yes

If applicable, is the statistical analysis and its interpretation appropriate?

Yes

Are all the source data underlying the results available to ensure full reproducibility?

Yes

Is the study design appropriate and is the work technically sound?

Partly

Are the conclusions drawn adequately supported by the results?

Yes

Are sufficient details of methods and analysis provided to allow replication by others?

Yes

Reviewer Expertise:

circadian rhythms, cancer

I confirm that I have read this submission and believe that I have an appropriate level of expertise to confirm that it is of an acceptable scientific standard.

# Peer review: Reviewer response for version 2

**DOI:** 10.5256/f1000research.153932.r193308

**Published:** 2023-08-09

**Authors:** Tianpeng Zhang

**Affiliations:** 1 Guangzhou University, Guangzhou, Guangdong, China

Authors have responded my comments.

Is the work clearly and accurately presented and does it cite the current literature?

Yes

If applicable, is the statistical analysis and its interpretation appropriate?

Yes

Are all the source data underlying the results available to ensure full reproducibility?

Yes

Is the study design appropriate and is the work technically sound?

Yes

Are the conclusions drawn adequately supported by the results?

Yes

Are sufficient details of methods and analysis provided to allow replication by others?

Partly

Reviewer Expertise:

Circadian clock and metabolism

I confirm that I have read this submission and believe that I have an appropriate level of expertise to confirm that it is of an acceptable scientific standard.

# Peer review: Reviewer response for version 1

**DOI:** 10.5256/f1000research.150145.r184831

**Published:** 2023-08-11

**Authors:** Brian J. Altman

**Affiliations:** 1 Department of Biomedical Genetics, Wilmot Cancer Institute, University of Rochester Medical Center, Rochester, NY, USA

In “The impact of shift-work light conditions on tissue-specific circadian rhythms of canonical clock genes: insights from a mouse model study”, Koritala, Dakup, and colleagues explore the outcome of short- and long-term exposure to rotating shift work compared to normal conditions and jet lag in circadian rhythms of the skin and liver in mice.  This aims to fill a knowledge gap where only short-term shift work experiments have been conducted on humans in laboratory settings, whereas human shift workers tend to engage in rotating shift work for many years.  The Authors find that, in general, short-term shift work has a subtle effect on rhythmicity of molecular clock gene expression, particularly in the liver.  In longer-term shift work and jet lag, the *Cry *genes were the most sensitive to disruption; however, long-term shift work and jet lag both dramatically affected the oscillatory characteristics of many circadian genes, often in a disparate fashion.  The Authors conclude that different rotating work and sleep schedules can have outsize impacts on molecular clock oscillation that may vary between tissues.

 Overall, this is a well-designed and executed study with clear outcomes and interpretable results. I have several minor suggestions and comments that I believe will enhance the quality of the final manuscript.

1. This study was only carried out in male mice, which goes against the NIH policy “Consideration of Sex as a Biological Variable in NIH-funded Research” (NOT-OD-15-102).  The Authors should discuss this in their Limitations section, and how the outcomes may be different in females.
2. In Figure 4, it is difficult to assess which oscillation characteristics are significantly different from control just by looking at the figure.  I am aware that full statistics are available in Extended Data Table 1, but it would be best if the Authors also figured out a way to represent, perhaps with a different shape (a star instead of a circle?) which datapoints were significantly different in Figure 4.
3. In the Discussion, the Authors mainly focus on the observed differences between short-term and long-term rotating shift work, which is indeed important.  Equally important is the fact that many papers and groups use the jet lag protocol as a proxy for shift work, and the Authors demonstrate here that the outcomes of jet lag can be quite different from either short-term or long-term rotating shift work.  This is a very important finding that should be addressed in the Discussion.

Is the work clearly and accurately presented and does it cite the current literature?

Yes

If applicable, is the statistical analysis and its interpretation appropriate?

Yes

Are all the source data underlying the results available to ensure full reproducibility?

Yes

Is the study design appropriate and is the work technically sound?

Partly

Are the conclusions drawn adequately supported by the results?

Yes

Are sufficient details of methods and analysis provided to allow replication by others?

Yes

Reviewer Expertise:

Molecular cancer biology, lung biology, immunology, circadian rhythms, metabolism

I confirm that I have read this submission and believe that I have an appropriate level of expertise to confirm that it is of an acceptable scientific standard.

# Peer review: Reviewer response for version 1

**DOI:** 10.5256/f1000research.150145.r184816

**Published:** 2023-07-13

**Authors:** Tianpeng Zhang

**Affiliations:** 1 Guangzhou University, Guangzhou, Guangdong, China

In this manuscript, Koritala *et al.* report that shift-work light affects the circadian rhythms of canonical clock genes in the liver and skin of mice. Although the authors have utilized multiple approaches to test their hypothesis, there remain areas where the methods section should also provide detailed information. Please refer to the following comments for more details.

1. Describe in detail the process for collecting skin, which part of the skin tissue was collected?
2. Is the selection of light intensity reasonable in the experiment? Multiple studies have shown that bright light can affect the circadian rhythm of activity, metabolism and clock gene expression (Bano-Otalora *et al.,* 2021 ^1^; Alves-Simoes *et al.*, 2016 ^2^; Fan *et al.*, 2022 ^3^). Meanwhile, the light intensity also affects the impact of chronic jet-lag.
3. More information should be provided on Circadian rhythm analysis, especially the setting of parameters.
4. Why use two housekeeping genes in the same tissue for clock gene relative quantification?
5. The author should discuss why *Cry* genes are preferentially altered under three light conditions.

Is the work clearly and accurately presented and does it cite the current literature?

Yes

If applicable, is the statistical analysis and its interpretation appropriate?

Yes

Are all the source data underlying the results available to ensure full reproducibility?

Yes

Is the study design appropriate and is the work technically sound?

Yes

Are the conclusions drawn adequately supported by the results?

Yes

Are sufficient details of methods and analysis provided to allow replication by others?

Partly

Reviewer Expertise:

circadian clock and metabolism

I confirm that I have read this submission and believe that I have an appropriate level of expertise to confirm that it is of an acceptable scientific standard, however I have significant reservations, as outlined above.

## References

[1] : Bright daytime light enhances circadian amplitude in a diurnal mammal. *Proc Natl Acad Sci U S A* .2021;118(22) : 10.1073/pnas.2100094118 10.1073/pnas.2100094118 34031246PMC8179182

[2] : Effects of type of light on mouse circadian behaviour and stress levels. *Lab Anim* .2016;50(1) : 10.1177/0023677215588052 21-9 10.1177/0023677215588052 25979911

[3] : Light intensity alters the effects of light-induced circadian disruption on glucose and lipid metabolism in mice. *Am J Physiol Endocrinol Metab* .2022;322(1) : 10.1152/ajpendo.00025.2021 E1-E9 10.1152/ajpendo.00025.2021 34719945
